# Influence of hydrostatic gradients and diurnal rhythm on cerebral and ocular blood flow

**DOI:** 10.1007/s00421-025-06038-z

**Published:** 2025-12-19

**Authors:** Carmen Possnig, Hendrik Mugele, Ronja Mittermeier, Justin S. Lawley

**Affiliations:** 1 Department of Sport Science, Division of Performance Physiology and Prevention, University of Innsbruck, Innsbruck, Austria; 2 Institute of Mountain Emergency Medicine, Eurac Research, Bolzano, Italy

**Keywords:** Cerebral blood flow, Eye blood flow, SANS, Diurnal rhythm, LBNP

## Abstract

Vascular dynamics in the eyes and brain under microgravity have gained attention due to the Spaceflight-Associated Neuro-Ocular Syndrome (SANS). We explored how changes in gravitational vectors, circadian rhythm, and sleep regulate cerebral blood flow (CBF) and eye perfusion. Hypothesizing that the eye lacks the capacity to autoregulate, we expected increases in blood flow or volume to drive choroidal engorgement in SANS, and circadian and sleep-related changes to further influence these dynamics. Lower-body negative pressure (LBNP) was investigated as a countermeasure for posterior ciliary artery velocity (PCAv) changes. 16 participants were examined in seated, supine, lateral, and prone positions to assess blood flow in the internal carotid and vertebral artery, and PCAv and middle cerebral artery velocity (MCAv) via ultrasonography. CBF and PCAv were stable in-between horizontal positions, and both lowest in a seated position: CBF (supine, 1036 ± 231 vs. seated, 889 ± 177 ml∙min^− 1^; *P* = 0.0019), PCAv (supine, 7.24 ± 1.8 vs. seated, 5.46 ± 1.3 cm∙sec^− 1^; *P* = 0.0012). CBF conductance was lower in the seated position (*P* = 0.0303), but after correcting for intraocular pressure and hydrostatic columns, PCA conductance remained stable (*P* = 0.0876). Oxygen delivery was higher in the supine position (supine, 212 ± 54 vs. seated, 182 ± 41 ml∙min^− 1^; *P* = 0.0022), but unchanged between horizontal positions (both *P* > 0.29). No diurnal changes were observed (all *P* > 0.15). Sleep decreased MCAv (*P* < 0.0001), heart rate (*P* = 0.0019), and mean arterial pressure (*P* = 0.0019). LBNP unexpectedly increased PCAv (*P* = 0.0358) and ocular perfusion pressure (*P* = 0.0156). In conclusion, CBF and PCA velocity change similarly with postural changes on Earth, and low-level LBNP was ineffective in lowering PCAv.

## Introduction

Spaceflight imposes unique challenges to the human cardiovascular system, which is finely tuned to our planet’s environmental conditions. In particular, a combination of changes in gravity (Garrett et al. 2017; Jacob et al. 2005; Lawley et al. 2017; Ogoh et al. 2016), changes in circadian biology (Diamant et al. 2002; Morris et al. 2013; Webb et al. 2024) and sleep (Gundel et al. 1997; Klingelhöfer 2012; Klingelhöfer et al. 1995; Kuboyama et al. 1997) may be implicated in the development of novel syndromes such as the Spaceflight-Associated Neuro-Ocular Syndrome (SANS).

SANS is characterized by optic disk edema, globe flattening, choroid engorgement and visual impairment (Lee et al. 2018; Mader et al. 2011). At present the pathophysiology of SANS is unknown but hypotheses include an increase in intracranial pressure (ICP) relative to the 24-hour average value observed on Earth (Iwasaki et al. 2021), vascular adaptions to microgravity (e.g. increased venous congestion reducing ocular outflow (Harris et al. 2022), and/or interactions of vascular, cerebrospinal and glymphatic fluid systems implicating blood flow regulation to the brain and the eyes.

One major limitation in identifying the pathophysiology of SANS is classifying which hemodynamic adaptations to spaceflight are different from, and theoretically maladaptive to, those typically observed on Earth. For example, Earth-based studies classically characterize physiological systems in the awake supine posture, and by convenience, experiments are often performed in the morning hours (~ 9 a.m.) despite hypothetical diurnal variations in many physiological systems. Humans typically spend 2/3^rds^ of the day in the upright posture while awake, and when lying down are generally asleep in either the supine, lateral or prone position. In contrast, during space flight, there are no gravitational gradients and due to cephalad fluid shifts, astronauts are closer to the supine and/or slightly head-down tilt position for the duration of the mission.

Previously several studies have noted that cerebral blood flow is influenced by postural changes, particularly decreasing in an upright compared to horizontal position (Garrett et al. 2017). Blood flow to the eye is more variable, with studies investigating postural changes from seated to supine seeing increases (Longo et al. 2004), decreases (Kaeser 2005), or stable flow (Baxter et al. 1992). Choroidal volume tends to increase with the supine posture (compared to a seated posture), while choroid thickness especially increases in the prone position (Anderson et al. 2017).

In regards to changes throughout the day, several studies have attempted to examine diurnal variations in cerebral blood flow, but a recent review (Webb et al. 2024) highlighted that this data is both conflicting and could be improved by more advanced quantitative measurements of cerebral blood flow. While there is clear data indicating that choroidal tissue varies in thickness throughout the day (Lee et al. 2014; Ostrin et al. 2023), peaking in the late evening or early nighttime (Nickla 2006; Nickla et al. 2002), to our knowledge, there are no studies characterizing vascular haemodynamics to the eye over a typical day. This is important as studies with chicks and guinea pigs have shown correlations between increased choroidal blood flow and thickness (Ostrin et al. 2023).

Another important factor is the impact of sleep. CBF has been observed to decrease particularly in slow-wave stages and during the early night (Klingelhöfer 2012; Klingelhöfer et al. 1995; Kuboyama et al. 1997), however how this relates to typical changes in posture (supine, prone, lateral) and including the upright posture is important to characterize a “typical” 24 h period.

It is clear that we have a limited understanding of how typical “gravitational-stressors” and daily variations in posture impact several important features of the cardiovascular system related to SANS. Therefore, the first aim of this study was to document changes in blood flow and vascular conductance of the cerebral arteries and the posterior ciliary artery during typical changes in gravitational gradients experienced on Earth. (1) When changing from the upright to the supine posture (Gx-axis) while awake and (2) while rotating around the z-axis (supine, lateral, prone) while awake. The second aim was to examine any diurnal variations in these two parameters over a typical day (9 a.m. – 5 p.m.) excluding the waking period where blood pressure typically surges relative to a period of sleep (Diamant et al. 2002; Veerman et al. 1995). The third aim was to continuously monitor changes in blood flow alongside powerful regulators of CBF (arterial blood pressure and end-tidal CO_2_) throughout a period of sleep. Finally, during the design of this study, we published several studies documenting that daily or nightly LBNP reduces choroidal engorgement associated with short-term bed rest (Hearon et al. 2022; Lawley et al. 2020). Thus, we developed a fourth aim to identify if low-level LBNP causes a reduction in PCAv which might explain the reduction in choroidal volume (Ostrin et al. 2023).

## Materials and methods

### Ethical approval

All participants were informed of the purpose and risks of each procedure and signed an informed consent form, which was approved by the Institutional Review Board at the University of Innsbruck and followed guidelines set forth in the Declaration of Helsinki.

### Participants

12 men (age 29 ± 4 yrs; height 179 ± 6 cm; weight 75 ± 7 kg) and 4 women (age 28 ± 3 yrs; height 171 ± 4 cm; weight 65 ± 5 kg) took part in study 1. In study 2, 4 men (age 34 ± 4 yrs; height 180 ± 5 cm; weight 86 ± 11 kg) and 4 women (age 31 ± 3 yrs; height 171 ± 4 cm; weight 64 ± 8 kg) participated.

All participants were healthy, non-smokers and free from cardiovascular, metabolic, and neuromuscular diseases. Female participants were tested during their self-reported early follicular phase (Sims and Heather 2018) for study 1. Food intake was not controlled over the 24 h, but participants were asked to eat the same breakfast before each morning (9 a.m.) measurement. Participants refrained from doing strenuous exercise or drinking beverages containing alcohol or caffeine 24 h before and during both studies.

### Experimental procedures

#### Protocol: study 1

All trials were completed in a quiet, climate-controlled environment in the Institute for Sports Science at the University of Innsbruck. Measurements were taken in the seated and supine position on day 1 at 9 a.m. and 5 p.m. to examine potential diurnal variations. At 9.a.m. measurements were obtained in the seated, supine, lateral and prone position, the order of which was randomized, on a standard hospital bed to examine the effect of posture. Participants then spent the night at the laboratory where a select set of measurements were obtained during sleep.

After a 20-minute rest phase to allow for a steady-state (~ 10 min for in-between lying positions), we measured middle cerebral artery velocity, as well as internal carotid (ICA) and vertebral artery (VA) blood flow. End-tidal CO_2_ (EtCO_2_), blood pressure (finger and brachial artery cuff pressure) and heart rate were measured continuously, and choroid artery blood flow was estimated via Doppler ultrasound of the posterior ciliary artery velocity through the right closed eye. A haemoglobin sample was taken from earlobe capillaries.

During the night, participants were equipped with a transcranial Doppler ultrasound probe attached to a headset (left MCA), electrocardiogram (heart rate), nasal cannula (EtCO_2_) and a finger cuff (continuous blood pressure measurements). The sleeping trial was started at each participant’s usual bed-time, and stopped upon waking the next morning. Mean time asleep was 7 h. Participants gave verbal feedback when going to sleep and upon waking up. Night-time measurements were divided into the following segments, starting with sleep onset: hours 0–2, hours 2–4, hours 4–6, and hour 6-wake up time, as well as an overall mean over the time asleep.

#### Protocol: study 2

After 20-minute resting in the supine position, baseline measurements were taken: middle cerebral artery velocity, end-tidal CO_2_, heart rate and blood pressure. Thereafter, posterior ciliary artery velocity was measured. Participants were lying in a custom-made semi-airtight chamber sealed at the level of the iliac crest. Using a standard vacuum pump, pressure inside the chamber was then reduced to −20mmHg. The same measurements were repeated 10 min after onset of lower-body negative pressure.

#### Cardiorespiratory parameters

##### Heart rate

 Continuous recordings of heart rate were determined from a 3-lead electrocardiogram (Tram-rac, Solar 8000 M GE, Marquette, USA). *End-tidal carbon dioxide.* Exhaled carbon dioxide was sampled continously by capnography (Moxus CD-3 A Carbon Dioxide Analyzer, AEI Technologies Inc, USA) from a nasal cannula (Omnistream, Smart OmniLine CO2 oral/nasal sampling set, Oridion Medical, Israel) for determination of end-tidal CO_2_. *Arterial blood pressure.* Arterial blood pressure was continuously measured via a finger cuff (Finapres Nova, Finapres Medical Systems, The Netherlands), as well as being measured in duplicate by electrosphygmomanometry (Tango, SunTechMedical Instruments Inc., USA) with a microphone placed over the brachial artery to detect Korotkoff sounds, and averaged. For the finger cuff measurements, a height corrector was used to get mean arterial pressure (MAP) at heart level. *Haemoglobin*. Haemoglobin concentration was collected from ear capillaries (HemoCue Hb 201 + System, HemoCue AB, Sweden).

#### Cerebral arterial blood flow

##### Internal carotid and vertebral arteries

 The internal carotid and vertebral arteries were imaged using a 15 − 4 MHz linear-array Doppler probe (model 15L4A Smart Mark, uSmart 3300 NexGen, Terason, USA). All participants were measured on the left side, except for 1 participant due to high carotid sinus on the left side. Duplex mode was used to measure the time-averaged mean velocity via a Doppler audio transformer (pulse-wave mode with 60° insonation angle, Penn State, Hershey, Pennsylvania, USA)(Herr et al. 2010) over 5 min and, subsequently, artery diameter (B-mode) was measured over 30 s via a custom-made wall-tracking software. The algorithm used to measure diameter has previously been validated (Coolbaugh et al. 2016) and used by our group to measure changes in arterial diameter associated with exercise (Amin et al. 2021).

We assumed a bilateral symmetry of ICA and VA; therefore, total cerebral blood flow was calculated as the product of the left ICA and VA mean blood flow velocity (cm∙s^− 1^) and artery cross-sectional area (cm²) multiplied by two and expressed as millilitres per minute (ml∙min^− 1^).

##### Middle cerebral artery velocity

 We continuously recorded MCA velocity using transcranial Doppler ultrasound (Spencer Technologies ST3, Washington, USA). To locate the right middle cerebral artery, the sample volume was initially placed at a depth of 50 mm while performing a transcranial searching pattern. Simultaneous use of M- and Doppler modes helped identification of the correct artery. Optimal scanning depth was chosen based on signal morphology and velocity. The probe was then securely attached to the cranium by a headset, thanks to which its exact position and angle was preserved during repeated testing.

##### Posterior ciliary artery velocity

 Blood velocity in the posterior ciliary artery was measured using a 9-MHz linear-array Doppler probe (iE33; Philips, The Netherlands) positioned on the closed right eyelid with an insonation angle of 0°. Position and depth of the chosen insonation site were noted to ensure scanning of the same artery at every measurement (see Fig. 4 for an example of an ultrasound image and velocity trace).

All variables were recorded as analogue data and imported into LabChart (version 8.1.16).

## Calculations

### Arterial conductance

Arterial conductance was calculated as organ specific blood flow divided by the corresponding transmural pressure. Vascular conductance is calculated as it reflects changes in vascular tone after accounting for change in transmural pressure (Joyce et al. 2019; Lautt 1989). When attempting to identify why blood flow to the eye or brain changes in relation to different postures, it is important to consider each hydrostatic gradient and tissue pressure (Buckey 2001; Buckey et al. 2022), and adjust the calculation of conductance accordingly. For example, to adequately interpret blood velocities within the eye, ocular perfusion pressure as well as intraocular pressure needs to be integrated to understand the downstream tone of the eye’s vasculature (see Table 1).

**Table 1:** Arterial pressure corrections used for calculation of perfusion pressures

	Seated	Supine	Lateral	Prone
MAP_heart_ (mmHg)	93 ± 8	83 ± 8	84 ± 11	81 ± 6
ICP (mmHg)^1^	0.23	15.03	(15.03)	(15.03)
Hydrostatic column (L_heart_ – L_EAM_) (mmHg)	−26.59 ± 1.4	0	0	0
MAP_brain_ (mmHg)^2^	66 ± 7	68 ± 8	69 ± 11	66 ± 6
Hydrostatic column (L_EAM_ – L_PLC_) (mmHg)	0	−3.89	0	3.89
MAP_eye_ (mmHg)^3^	66 ± 7	79 ± 8	84 ± 11	85 ± 6
IOP (mmHg)^4^	11.5	13.7	(13.7)	20.3
OPP (mmHg)^5^	55 ± 7	65 ± 8	70 ± 11	65 ± 6

*MAP* Mean arterial pressure, *ICP* Intracranial pressure, *IOP* Intraocular pressure, *OPP* Ocular perfusion pressure, *L* Length, *EAM* External auditory meatus, *PLC* Posterior side of the lamina cribrosa

^1^(Petersenet al., 2018), values for seated and supine postures only but assumed to be equal in all horizontal positions

^2^MAP_heart_ minus ICP and (L_heart_ – L_EAM_) difference

^3^MAP_heart_ plus/minus (L_EAM_ – L_PLC_) difference and (L_heart_ – L_EAM_) difference

^4^(Andersonal, 2015), values for seated, supine, prone; lateral assumed to be equal to supine position

^5^MAP_eye_ minus IOP

### Cerebral vascular conductance

To calculate conductance at brain level (CBF conductance), blood flow through the ICA and/or VA was divided by cerebral perfusion pressure *(MAP - ICP).* MAP was measured at the brachial artery in all postures, but in the seated position corrected for the posture-dependant hydrostatic gradient between the arm and brain. The hydrostatic distance was individually measured as the length (cm) between the heart (position of Finapres height corrector) and external auditory meatus (EAM) and the following equation was used in the upright posture:

where ρ is the density of blood (1.06 g∙cm^− 3^), and *g* equals the gravitational acceleration (980.665 cm∙s^− 2^). (ρ*g*h) is calculated as dyne∙cm^− 2^, which was converted to the more practical unit of mmHg (1mmHg = 1333.22 dyne∙cm^− 2^), therefore.

ICP was not measured, but values were taken from observations in the literature using direct invasive recordings. The values for supine ICP, 15.03 mmHg, and upright ICP, 0.23 mmHg, were taken from (Petersen et al. 2018).

### Choroid vascular conductance

To calculate conductance in the eye, posterior ciliary artery velocity was divided by optic perfusion pressure *(MAP*_*brain*_ *- IOP)*. In addition, at eye level the hydrostatic column between the ear and the eye must be taken into account for correct arterial pressure, as well as intraocular pressure, in the supine, prone and lateral position (Anderson et al. 2016).

The following equations were applied to each posture:

Upright posture

$$MAP_{{eye}} = {\text{ }}MAP_{{EAM}} ~$$

Prone posture

$$MAP_{{eye}} = {\text{ }}MAP_{{EAM}} ~ + {\text{ }}\left[ {0.778*{\text{ }}\left( {L_{{EAM}} - {\text{ }}L_{{PLC}} } \right)} \right]$$

where L_PLC_ is the posterior side of the lamina cribrosa and estimated as located in **5 cm** distance to the external auditory meatus (Anderson et al. 2016). The relationship between cmH_2_O and mmHg depends on the density of the fluid (*1 cmH*_*2*_*O = (ρH*_*2*_0/ρHg) * 1 mmHg), therefore 0.778 is the conversion factor derived based on the relative densities of mercury (13.5951 g∙cm^− 3^) and blood (1.06 g∙cm^− 3^).

Supine posture

$$MAP_{{eye}} = {\text{ }}MAP_{{EAM}} ~ - {\text{ }}\left[ {0.778*{\text{ }}\left( {L_{{EAM}} - {\text{ }}L_{{PLC}} } \right)} \right]$$

Lateral posture

$$MAP_{{eye}} = {\text{ }}MAP$$

IOP was not measured, but values were taken from observations in the literature using applanation tonometry: Supine IOP, **13.7** mmHg, upright IOP, **11.5** mmHg, lateral IOP, **13.7** mmHg (as supine), prone IOP, **20.3** mmHg (Anderson et al. 2016).

### Conductance during application of LBNP

LBNP has an influence on both ICP and IOP. In line with data from a study by Petersen and colleagues, we adjusted ICP used for the calculation of MCAv conductance from the supine 15.03 mmHg to 11.79 mmHg with − 20 mmHg of LBNP (Petersen et al. 2018). Cerebral perfusion pressure was then calculated as above (MAP - ICP) for the baseline and LBNP respectively.

For the calculation of PCAv conductance, we used IOP levels from Hall and colleagues, who reported IOP for females and males separately. We used a mean of these two (20.08 mmHg for the supine baseline, 16.72 mmHg for LBNP) to calculate ocular perfusion pressure (MAP - IOP) and with this, PCAv conductance.

### Statistical analysis

Study 1: To examine the diurnal effect, haemodynamic data was compared between 9 a.m. and 5 p.m. with a paired t-test. The effect of posture was analysed using one-way repeated measures (seated, supine, prone, lateral, at 9 a.m. only) ANOVAs with a post-hoc Holm-Šídák multiple comparisons test to determine haemodynamic responses between the seated and supine, lateral and prone posture. The effect of sleep was analysed using one-way repeated measures (supine (5 p.m.) vs. 2, 4, 6 and 8 h of sleep and a mean over all sleep time points) in a mixed-effects analysis (one participant woke up after 6 h and therefore values were missing for the 6-awake time point) with a post-hoc Holm-Šídák multiple comparisons test to determine haemodynamic responses. Study 2: A paired t-test (baseline vs. LBNP) was used to determine the effect of LBNP.

All values are expressed as mean ± standard deviation. Normality of the data was assured using a Shapiro-Wilks test. All statistical procedures were completed on Prism version 10 (GraphPad, FL, USA) where statistical significance was set to *P* ≤ 0.05.

## Results

### Postural changes

#### Cerebral blood flow

 Total cerebral blood flow was higher in the horizontal positions, with statistically significant changes between the seated and supine posture (seated, 889 ± 177, vs. supine, 1036 ± 231 ml∙min^− 1^; *P* = 0.0019). No significant difference was found between the horizontal positions (supine vs. lateral, 1012 ± 174 ml∙min^− 1^; *P* = 0.5735; supine vs. prone, 972 ± 237 ml∙min^− 1^, *P* = 0.2900). Cerebrovascular conductance was lower in the seated compared to the supine position (seated, 13.6 ± 3.2, vs. supine, 15.5 ± 3.7 ml·min^− 1^·mmHg^− 1^, *P* = 0.0303) but not changed in-between horizontal positions (both *P* = 0.6861). Similarly, middle cerebral artery velocity changed between the supine and seated (seated, 49.4 ± 6.9 vs. supine, 52.55 ± 7.7 cm∙sec^− 1^; *P* = 0.0315), but not the supine and lateral positions (supine vs. lateral, 53.2 ± 7.5 cm∙sec^− 1^; *P* = 0.6332), nor between the supine and prone position (supine vs. prone, 53.9 ± 10.3 cm∙sec^− 1^; *P* = 0.5756). Oxygen delivery was significantly higher in the supine compared to the seated position (seated, 182 ± 41, vs. supine, 212 ± 54 ml∙min^− 1^; *P* = 0.0022), but not between supine and lateral (lateral, 204 ± 41 ml∙min^− 1^; *P* = 0.3851), or the prone position (prone, 198 ± 48 ml∙min^− 1^; *P* = 0.2957).

#### Blood velocity in the posterior ciliary artery

 Velocity in the posterior ciliary artery was lower in the seated compared to the supine posture (seated, 5.46 ± 1.3 vs. supine, 7.24 ± 1.8 cm∙sec^− 1^; *P* = 0.0012), but not different between horizontal postures (supine vs. lateral, 7.17 ± 1.4 cm∙sec^− 1^; *P* = 0.8008; supine vs. prone, 6.31 ± 1.6 cm∙sec^− 1^; *P* = 0.0692).

**Fig. 1:**
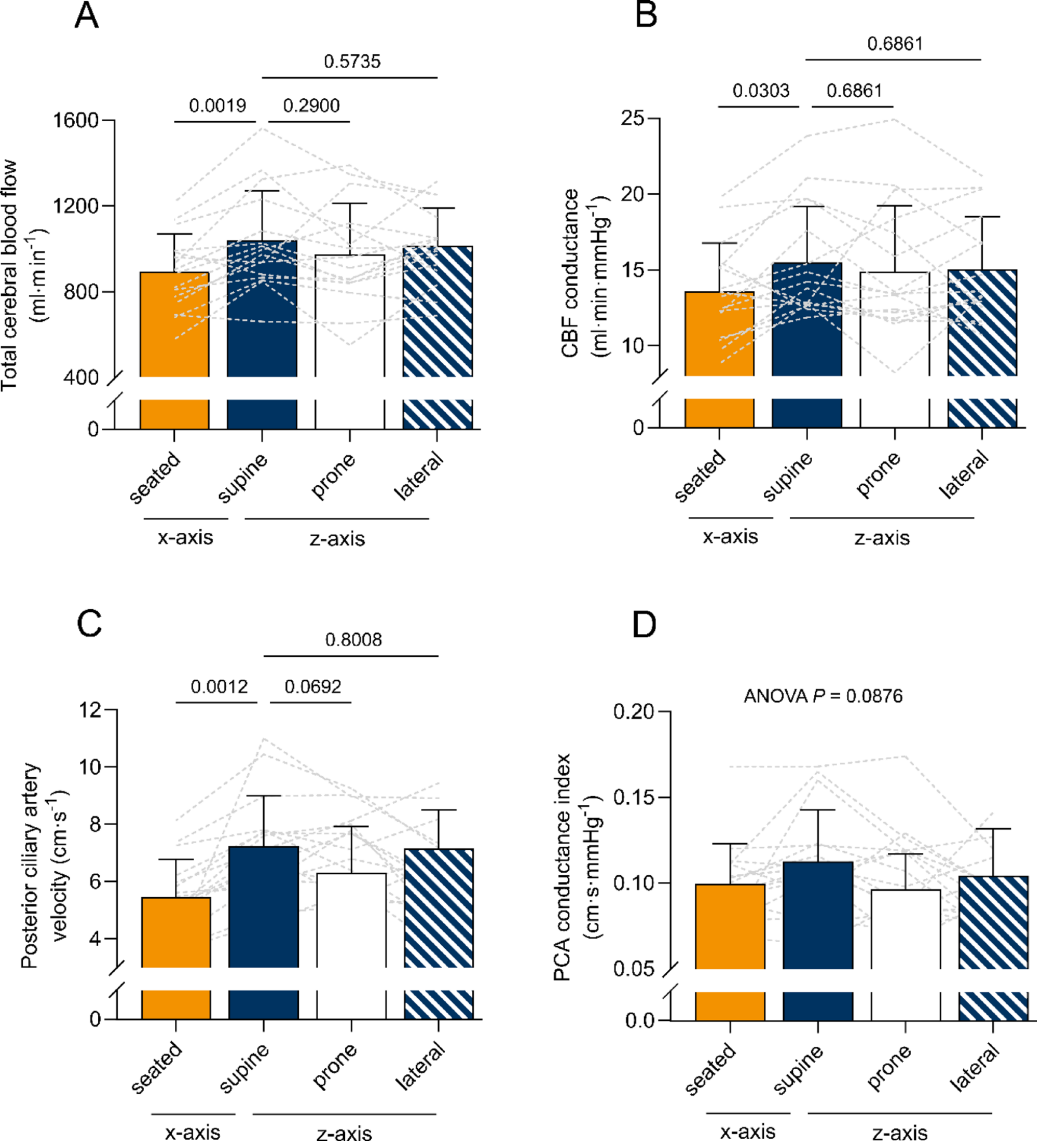
Changes in cerebral blood flow and posterior ciliary artery velocity over different positions

After correcting for the intraocular pressure and hydrostatic columns (see methods section), no difference was seen in posterior ciliary artery conductance in-between positions (*P* = 0.0876). An overview of changes in blood flow over different body positions is presented in Table 2; Fig. 1.

**Table 2:** Differences in systemic and cerebral hemodynamic parameters over 4 postures in the morning

	Seated	Supine	Lateral	Prone
Total CBF (ml·min^− 1^)	889 ± 177**	1035 ± 231	1012 ± 174	972 ± 237
CBF conductance (ml·min·mmHg^− 1^)	13.59 ± 3.2*	15.48 ± 3.7	15.04 ± 3.5	14.89 ± 4.3
Oxygen delivery (ml·min^− 1^)	183 ± 41**	212 ± 54	204 ± 41	198 ± 48
PCAv (cm·s^− 1^)	5.5 ± 1.3**	7.2 ± 1.8	7.2 ± 1.4	6.3 ± 1.6
PCA conductance index (cm·s·mmHg^− 1^)	0.099 ± 0.02	0.113 ± 0.03	0.105 ± 0.03	0.097 ± 0.02
MCAv (cm·s^− 1^)	49.4 ± 6.9*	52.6 ± 7.7	53.2 ± 7.5	53.9 ± 10.3
Hb (g·dl^− 1^)	15.51 ± 1.3*	15.23 ± 1.4	15.27 ± 1.4	15.25 ± 1.3
HR (beats·min^− 1^)	61 ± 9*	56 ± 8	57 ± 9	57 ± 10
EtCO2 (%)	4.6 ± 0.4*	4.8 ± 0.5	4.8 ± 0.5	4.9 ± 0.4

*P*-values are all compared to supine posture

*CBF* Cerebral blood flow, *PCAv* Posterior ciliary artery velocity, *MCAv* Middle cerebral artery velocity, *Hb* Hemoglobin, *HR* Heart rate, *EtCO*_2_, End-tidal CO_2_

*p<0.05 and ^**^p<0.01

#### Respiratory and cardiovascular parameters

 Heart rate was lower in the supine than the seated position (supine, 56 ± 8, vs. seated, 61.5 ± 9, beats∙min^− 1^, *P* = 0.0194), but not different in between horizontal positions (supine vs. lateral, 57 ± 9 beats∙min^− 1^, *P* = 0.594; supine vs. prone, 57.3 ± 10 beats∙min^− 1^, *P* = 0.594); and similar changes were visible for end-tidal CO_2_ which was lower in the seated compared to supine position, with a trend to increase slightly more in the prone compared to the supine position (supine, 4.8 ± 0.5, vs. seated, 4.63 ± 0.4%, *P* = 0.0225; supine vs. lateral, 4.76 ± 0.5%; *P* = 0.2546; supine vs. prone 4.91 ± 0.4%; *P* = 0.0562). Mean arterial pressure at brain level was higher in the supine than the seated posture (supine, 82.6 ± 8, vs. seated, 67.9 ± 7.1 mmHg, *P* < 0.0001) and unchanged in between horizontal positions (supine vs. lateral, 83.7 ± 10.6 mmHg, *P* = 0.634; supine vs. prone, 81.2 ± 6.5 mmHg, *P* = 0.634).

A slight haemoconcentration was seen in the upright position (seated, 15.5 ± 1.3 vs. supine, 15.23 ± 1.4 g∙dL^− 1^, *P* = 0.0331), while levels in the horizontal positions remained stable (lateral, 15.27 ± 1.4, prone, 15.25 ± 1.3 g∙dL^− 1^, both *P* = 0.9301).

**Fig. 2:**
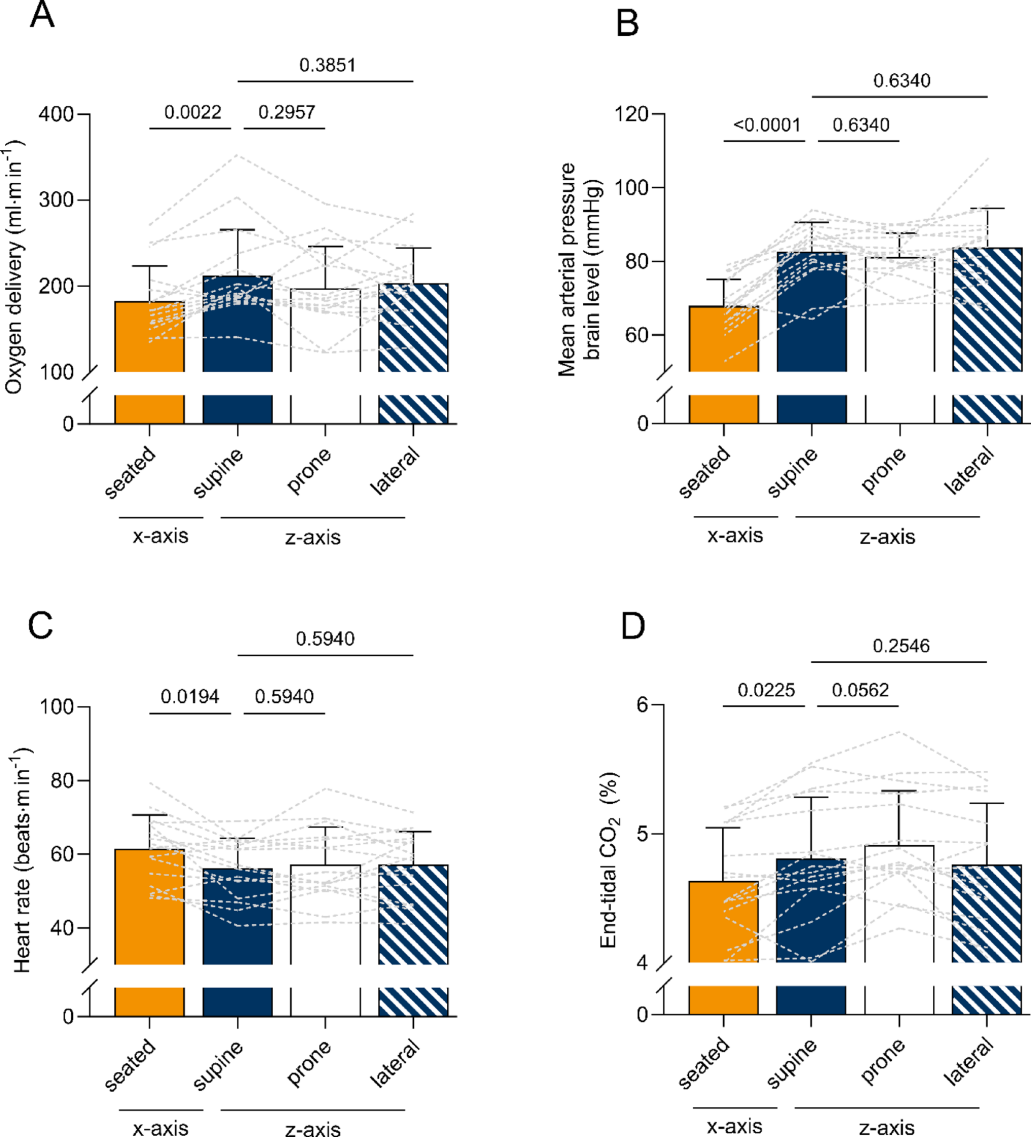
Changes in cardiovascular and respiratory parameters over different positions

An overview of changes of respiratory and cardiovascular parameters over different body positions is presented in Tables 1 and 2; Fig. 2.

### Diurnal changes in cerebral blood flow and haemodynamics

No significant diurnal variations were observed in CBF (all *P* values > 0.4), MCAv (all *P* values > 0.5), PCAv (all *P* values > 0.15), heart rate (all *P* > 0.21), or mean arterial pressure (all *P* > 0.75). Albeit total CBF in the seated position (*P* = 0.0822) and PCAv in the lateral position (*P* = 0.0803) showed a trend to statistical significance between morning and afternoon.

An overview of diurnal changes is presented in Table 3.

**Table 3:** Diurnal variations

			**Supine**		Seated		
			a.m.	p.m.	a.m.		p.m.
Total CBF (ml·min^− 1^)			1035 ± 231	1054 ± 179	889 ± 177		953 ± 182
CBF conductance (ml·min·mmHg^− 1^)			15.48±3.7	15.9 ± 3.4	13.59 ± 3.2		14.98 ± 3.8*
Oxygen delivery (ml·min^− 1^)			212 ± 54	210 ± 45	182 ± 41		195 ± 45
PCAv (cm·s^− 1^)			7.24 ± 1.8	7.28 ± 2.4	5.46 ± 1.3		6.31 ± 2.1
PCA conductance index (cm·s·mmHg^− 1^)			0.113 ± 0.03	0.1153 ± 0.05	0.0997 ± 0.02		0.1217 ± 0.05
MCAv (cm·s^− 1^)			52.6 ± 7.7	53.9 ± 8.3	49.4 ± 6.9		51 ± 8.7
HR (beats·min^− 1^)			56 ± 8	58 ± 7	62 ± 9		64 ± 11
MAP_brain_ (mmHg)^1^			68 ± 8	67 ± 8	66 ± 7		65 ± 8

*CBF* Cerebral blood flow, *PCAv* Posterior ciliary artery velocity, *MCAv* Middle cerebral artery velocity, *HR* Heart rate, *MAP* Mean arterial pressure

*p < 0.05

^1^MAP_heart_ minus ICP and (L_heart_ – L_EAM_) difference

### Changes over night

MCA velocity decreased overnight compared to the afternoon supine measurement in all time points (afternoon, 53.9 ± 8.3 vs. 0–2 h, 49.8 ± 9 cm∙sec^− 1^; *P* = 0.0262; afternoon vs. hours 2–4, 44.3 ± 7.7 cm∙sec^− 1^; *P* < 0.0001, afternoon vs. hours 4–6, 42.6 ± 7.8 cm∙sec^− 1^; *P* < 0.0001). MCAv increased again shortly before waking up (6-awake 47.4 ± 9.2 cm∙sec^− 1^; *P* = 0.00012). Moreover, the average night time value was significantly lower than the afternoon measurements (45.9 ± 8.2 cm∙sec^− 1^; *P* < 0.0001).

Mean arterial blood pressure decreased starting with hour 0–2 (afternoon, 83 ± 8 vs. 0–2, 75 ± 8 mmHg; *P* = 0.0027), stayed below the evening values in hours 2–4 (73 ± 7 mmHg; *P* = 0.0019), hours 4–6 (76 ± 7.2; *P* = 0.0089), and increased slightly before waking (79 ± 7 mmHg; *P* = 0.0539). The mean value over the whole night was also significantly lower (*P* = 0.0019). The cerebrovascular conductance index was decreased compared to afternoon supine posture only in the middle of the night (afternoon, 0.661 ± 0.12 vs. hours 4–6, 0.567 ± 0.11 cm∙sec^− 1^∙mmHg^− 1^; *P* = 0.0291). There was no significant difference in end-tidal CO_2_ over the night (*P* = 0.1012).

Heart rate varied over the period spent asleep. While heart rate did not change significantly during the first hours of the night (afternoon, 58 ± 7 vs. hours 0–2, 57 ± 7 beats∙min^− 1^; *P* = 0.8021), heart rate decreased starting from the 2nd hour of sleep (afternoon vs. hours 2–4, 53 ± 6 beats∙min^− 1^; *P* = 0.0227; vs. hours 4–6, 51 ± 7 beats∙min^− 1^; *P* = 0.0009; and 6-awake, 50 ± 7 beats∙min^− 1^, *P* = 0.0019). The all-night mean value of heart rate was decreased compared to supine levels (52 ± 7 beats∙min^− 1^, *P* = 0.0019).

**Fig. 3:**
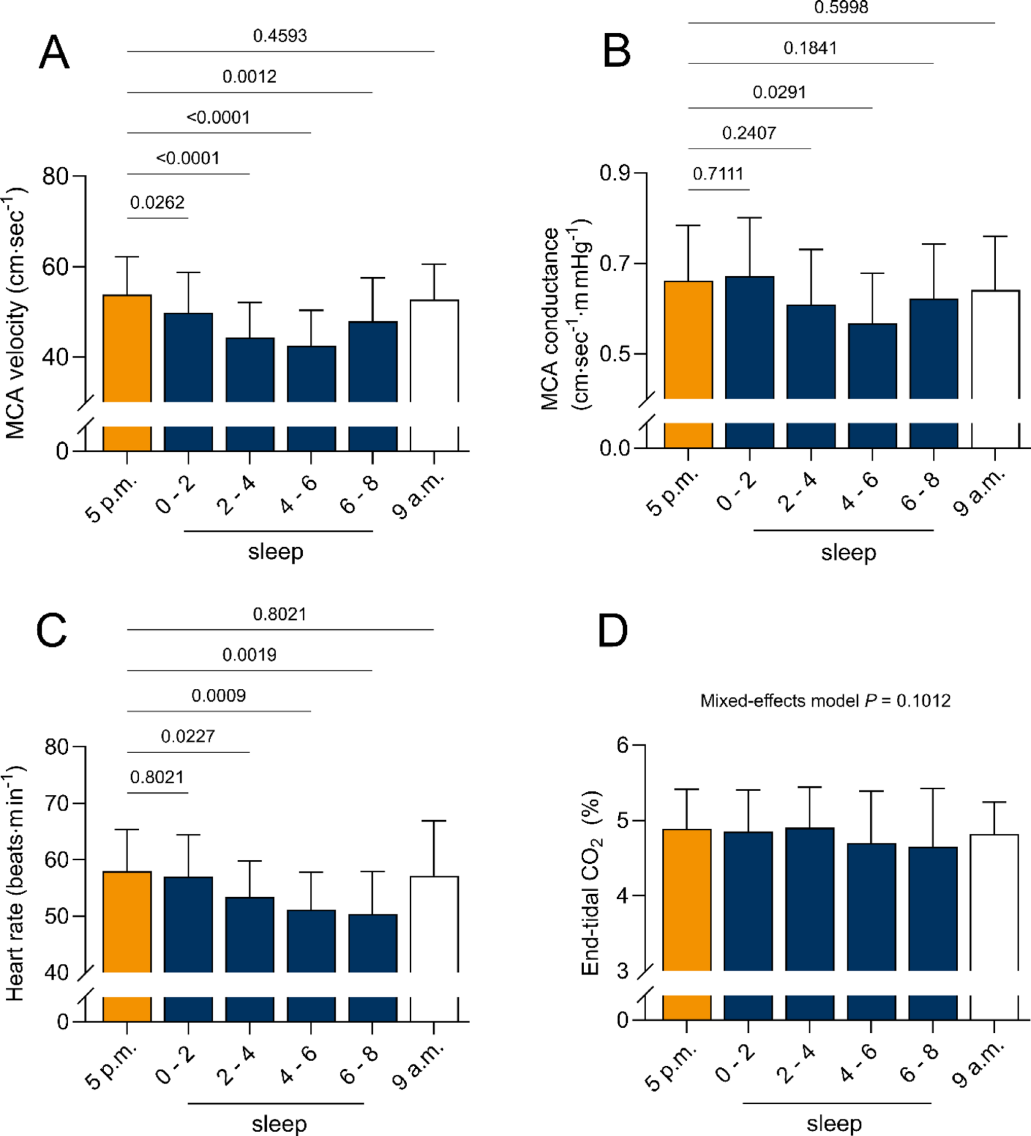
Changes in middle cerebral artery (MCA) velocity and conductance, heart rate and end-tidal CO2 during sleep

An overview of changes during sleep is presented in Table 4; Fig. 3.

**Table 4:** Nighttime changes

	Afternoon supine	0–2 h	2–4 h	4–6 h	6-awake	Mean night
MCAv (cm·s^− 1^)	53.9 ± 8.3	49.8 ± 8.9*	44.3 ± 7.7**	42.6 ± 7.8**	47.4 ± 9.2**	45.9 ± 8.2**
HR (beats·min^− 1^)	58 ± 7	57 ± 7	53.4 ± 6*	51.2 ± 7**	50.4 ± 7**	52.7 ± 7**
MAP_heart_ (mmHg)	82.3 ± 8	74.7 ± 7.6**	73.5 ± 7.3**	75.7 ± 7.2**	79.3 ± 8.5	75.8 ± 6.3**
CVCi (cm·s·mmHg^− 1^)	0.66 ± 0.12	0.67 ± 0.13	0.61 ± 0.12	0.57 ± 0.11*	0.6 ± 0.12	0.60 ± 0.11
EtCO2 (%)	4.89 ± 0.5	4.85 ± 0.6	4.9 ± 0.5	4.7 ± 0.7	4.65 ± 0.8	4.77 ± 0.5

*MCAv* middle cerebral artery velocity, *HR* heart rate, *MAP* mean arterial pressure, *CVCi* cerebrovascular conductance index, *EtCO*_*2*_ end-tidal CO_2_

*P*-values are all compared to afternoon supine measurement

*p < 0.05 and ^**^p < 0.01

### Effects of lower-body negative pressure

Low-level lower-body negative pressure increased posterior ciliary artery velocity (baseline, 7.33 ± 2.2, vs. LBNP, 7.98 ± 2.4 cm∙sec^− 1^, *P* = 0.0358). Ocular perfusion pressure, calculated with IOP values taken from (Hall et al. 2024), increased with LBNP (baseline, 40.1 ± 7 vs. LBNP, 45.5 ± 8 mmHg; *P* = 0.0454), while the PCA conductance index remained stable (*P* = 0.1825). In contrast, MCAv did not change (*P* = 0.7579), but the MCA conductance index decreased (baseline, 0.93 ± 0.18 vs. LBNP, 0.82 ± 0.16 cm∙sec^− 1^∙mmHg^− 1^; *P* = 0.0099). Mean arterial pressure at heart level did not change significantly (baseline, 71 ± 8 vs. LBNP, 77 ± 14 mmHg, *P* = 0.0856). No changes were seen for heart rate (*P* = 0.3827).

**Fig. 4:**
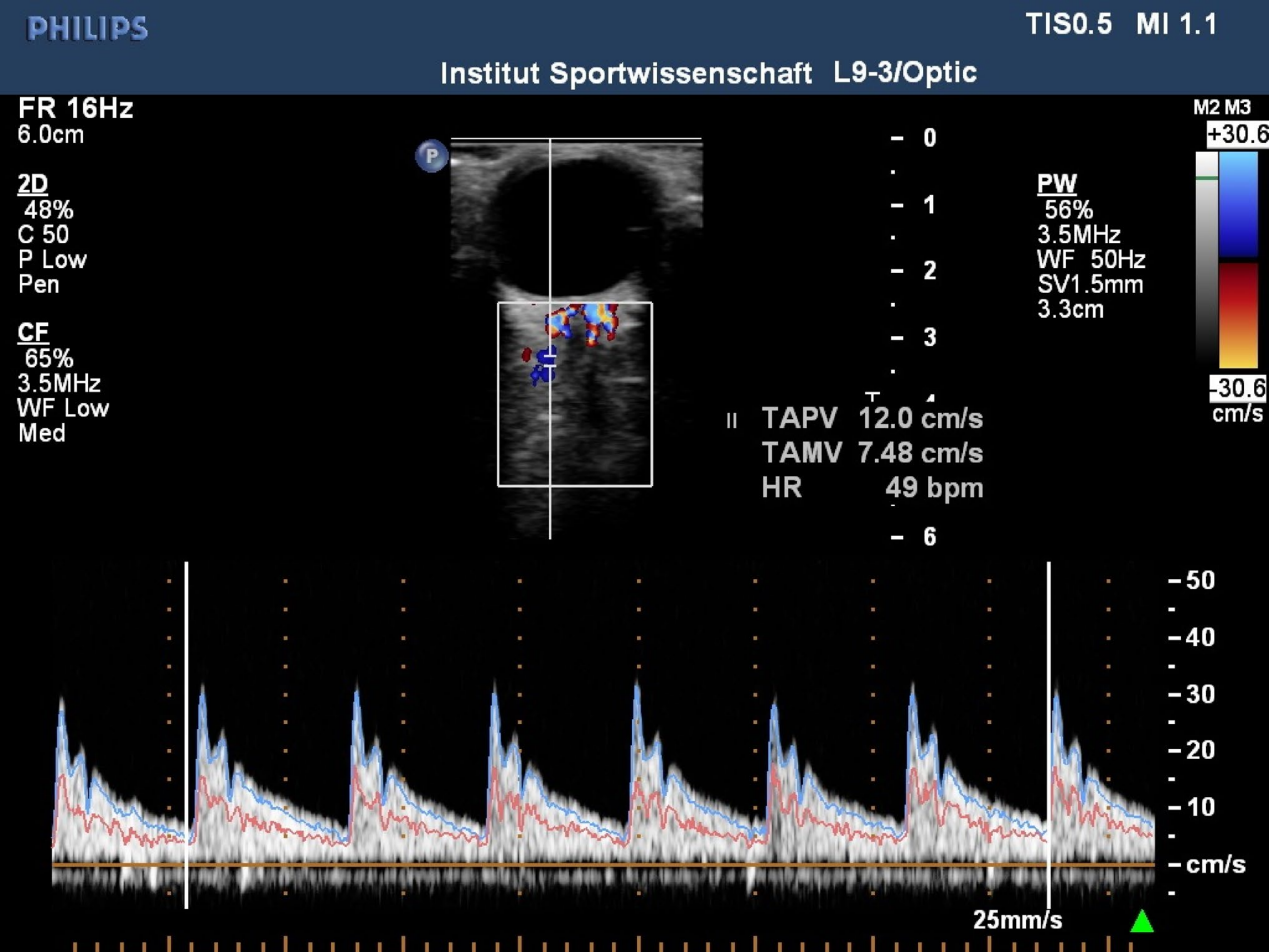
Example of PCAv ultrasound image and velocity trace

An overview of changes during LBNP application is presented in Table 5.

**Table 5:** Effects of lower-body negative pressure

	Baseline (0°)	LBNP (−20 mmHg)
PCAv (cm·s^− 1^)	7.33 ± 2.2	7.98 ± 2.4*
MAP_heart_ (mmHg)	70.9 ± 8	77 ± 14
ICP (mmHg)^1^	15.03	11.79
MAP_brain_ (mmHg)	55.9 ± 8	64.8 ± 14*
IOP (mmHg)^2^	20.8	16.72
OPP (mmHg)^3^	40.1 ± 7	45.5 ± 8*
PCA conductance index (cm·s·mmHg^− 1^)	0.144 ± 0.04	0.134 ± 0.04
MCAv (cm·s^− 1^)	51.4 ± 9	51.9 ± 11
MCA conductance index (cm·s·mmHg^− 1^)	0.93 ± 0.18	0.82 ± 0.16**
HR (beats·min^− 1^)	64 ± 7.4	66.1 ± 8.3

*PCAv* posterior ciliary artery velocity, *MAP* mean arterial pressure, *IOP* intraocular pressure, *OPP* ocular perfusion pressure, *MCAv* middle cerebral artery velocity, *HR* heart rate

*p < 0.05 and ^**^p < 0.01

^1^ (Petersenet al., 2018)

^2^ (Hall et al., 2024)

^3^ MAP_eye_ minus IOP

## Discussion

When comparing the effects of gravitational gradients on blood flow to the brain and blood velocity in the eye, four main findings were observed: (1) CBF and PCAv are sensitive to changes around the Gx-axis (supine vs. seated position), but not around the Gz axis (in-between horizontal positions). This was mirrored in MCA velocity, oxygen delivery, heart rate, mean arterial pressure, and end-tidal CO_2_. (2) Time of day had a limited effect on any of the hemodynamic variables studied. (3) MCA velocity decreased progressively overnight, while mean arterial blood pressure was consistently lower during sleep. However, the decrease in cerebrovascular conductance index highlights that vasoconstriction of the brain’s circulation is the major contributor to the fall in cerebral blood flow after the initial dip in blood pressure with sleep. (4) Application of low-level LBNP increased PCAv due to a slight increase in mean arterial pressure. Thus, PCA conductance index, as a marker of vascular volume, did not change.

### Effects of postural changes on cerebral blood flow

Blood flow to the brain was reduced in the upright versus supine posture, a finding that is consistent throughout the literature (Alperin et al. 2005; Garrett et al. 2017; Ogoh et al. 2016; Sato et al. 2012). The decrease in end-tidal CO_2_ in the seated position suggests that hypocapnia likely contributed to the reduction in cerebral blood flow and decreased oxygen delivery in the upright position. However, it is worth noting that while acute isocapnia prevents the fall in cerebral blood flow in the upright posture (Ogoh et al. 2016), cerebral blood flow transiently returns to seated values with prolonged isocapnia (Immink et al. 2009). In the prone position end-tidal CO_2_ was slightly higher (statistical trend) than in the supine posture. This is unexpected, as the prone position is a technique typically used to improve gas exchange in respiratory distress syndrome by removing weight on the chest and lessening lung compression (Aoyama et al. 2019). Why in the current study end-tidal CO_2_ was slightly elevated in the prone position is unknown (see Fig. 2; Table 2). Aas blood flow to the brain was unchanged around the Gz axis, this observation is mostly likely a type II statistical error or statistically correct, but with a 0.755 mmHg change in ETCO_2_ not of physiological significance. A 1 mmHg difference in end-tidal CO_2_ amounts to about 2–4% change in CBF (Willie et al. 2014). If we assume a 2% change in CBF for the 0.755 mmHg we observed, that would amount to 20.7 ml·min^− 1^, but importantly, we would expect the effect of increased end-tidal CO_2_ to be an increase in CBF. CBF however remained stable from the supine to prone position (1035 ± 231 vs. 972 ± 237 ml·min^− 1^), whereas end-tidal CO_2_ increased. An alternative explanation for the decrease in seated cerebral blood flow could be the mild hemoconcentration that was observed in the upright posture. Standing or sitting upright increases hydrostatic pressure in the lower body, leading to alterations in hydrostatic and oncotic pressures, which in turn promote the movement of blood plasma from the intravascular to the interstitial space (Krogh et al. 1932). Thus, haemoglobin concentration and arterial oxygen content are higher in the upright posture (Jacob et al. 2005; Tombridge 1968), whereby a lower cerebral blood flow would be expected to maintain oxygen delivery. Interestingly, we did not observe a full restoration of oxygen delivery in the upright posture (see Fig. 2; Table 2), indicating that hemoconcentration alone does not fully account for the reduction in cerebral blood flow. Yet, it is worth noting that the magnitude of hemoconcentration observed in the current study is smaller than typically observed in the literature and may suggest a technical limitation in calculating absolute values rather than a mechanistic insight. Future research manipulating both end-tidal CO_2_ and oxygen content is needed to tease out the mechanistic contributions.

### Effects of postural changes on eye blood flow

An increase in choroidal volume is a frequently observed phenomenon in bed rest studies and after spaceflight (Laurie et al. 2020; Mader et al. 2017). Although tissue remodeling may contribute to changes during prolonged spaceflight, an increase in choroidal volume has also been documented following short-term (3 days) bed rest (Hearon et al. 2022; Lawley et al. 2020), suggesting that more acute alterations in blood volume or water homeostasis may play a role. Prior studies have documented that the choroid has a limited capacity for autoregulation (Kaeser 2005), thus it could be expected that the eye is more sensitive to changes in gravitational gradients than cerebral blood flow. In the current study, PCAv and cerebral blood flow increased in the supine position compared to the upright position (see Fig. 1; Table 2). Initially, this observation might be attributed to downstream vasodilation of the choroid and cerebral vasculature. However, vascular conductance provides a more comprehensive measure of downstream vascular tone and an indirect assessment of blood volume dynamics than velocity or blood flow measurements alone. After accounting for posture-related changes in optic transmural pressure (i.e., mean arterial pressure at the brain level and intraocular pressure), vascular conductance of the PCA remained statistically unchanged (also numerically similar), while that of the brain clearly increased (see Table 1; Fig. 1). Therefore, it appears that changes in PCAv during changes in posture are predominantly due to an increase in ocular perfusion pressure rather than vasodilation of the choroid vasculature. Vice versa, there is a reflex change in arterial vascular tone in the cerebral circulation with a change in the Gx axis gravitational gradient.

### Effects of time of day

We observed no major effect of time of day for any of the data collected in the supine position (see Table 3). While there is substantial literature on diurnal variations in cardiorespiratory parameters, in particular for heart rate (De Scalzi et al. 1984), arterial blood pressure (Millar-Craig et al. 1978), and EtCO_2_ (Spengler et al. 2000; Stephenson et al. 2000), there is also a number of studies reporting an absence of a rhythm. For example, several investigations on shift workers have concluded that any daytime variations in blood pressure immediately adapt to shifted phases of activity and sleep and seem independent of internal synchronizers. Blood pressure was found to fluctuate during waking phases (be they during the day or during the night) in accordance with activity and postural changes (Baumgart et al. 1989; Pieper et al. 1993). To further examine a diurnal effect, Diamant and colleagues looked at daily variations in MCA velocity, heart rate, and blood pressure. During MCA velocity recordings, their participants lay in the supine position for 15min every hour, while the rest of the day was spent in upright seated positions. As heart rate and MAP were recorded continuously, results were analysed for both positions. No diurnal differences were found between supine measurements, but they did find some difference between daytime seated MAP, which was higher than nighttime (supine sleeping) MAP (Diamant et al. 2002). This variation however corresponds to the typical difference seen between a supine and a seated posture and is therefore not necessarily evoked by a circadian rhythm, but likely a result of posture and perhaps sleep. Sleep also had an effect on heart rate within the same study: while heart rate did not vary in any posture during daytime, it was lower at night compared to both seated and supine daytime values. This pattern is identical to what we observed in the present study. Diurnal variations were also investigated by Burma and colleagues (although without investigating changes during sleep). They examined their participants over several timepoints in an upright standing position and during exercise (squats). They did not observe any significant variations in heart rate, MAP, or EtCO_2_ in either the exercise or inactive condition (Burma et al. 2020). In summary, while sleep and changes in posture (i.e. upright versus supine) have prominent effects on heart rate and MAP, diurnal variations seem to have a minimal effect.

While we found differences between the awake and sleeping blood flow measurements in our study, none were observed between the morning and afternoon for cerebral blood flow, MCA velocity, or posterior ciliary artery velocity (see Table 3). There is substantial literature on variations of blood velocity (Webb et al. 2024) and arterial pressure independently (Degaute et al. 1991; Kawano 2011) during the day. However, the recent review by Webb and colleagues highlights the difficulty in obtaining adequate data to consider circadian variations in blood velocity and presents results with great variety (Webb et al. 2024). Differences in experimental setup and methods used likely contributed to contradictory outcomes: While some of the studies note that MCAv is lowest in the morning than in the afternoon, others report stable MCAv throughout the day. However, a more complete picture can be displayed by using magnetic resonance imaging (MRI). Indeed, a recent MRI study noted that there may be daytime variations even between different brain regions, observing that while neural activation and a blood oxygen level-dependent signal are decreased in the afternoon compared to mornings for some brain regions, the opposite happens in other regions (Jiang et al. 2016). Diamant and colleagues also observed time-of-day variations for MCAv in the supine position, with the lowest values in the morning at around 10 a.m., and the highest in the afternoon at around 6 p.m., thus corresponding to the two time points our measurements were taken, although not matching our results.

### Effects of sleep

Measuring middle cerebral artery velocity via a transcranial Doppler, we found progressive decreases in MCAv and MCA conductance during sleep compared to the supine awake state (see Fig. 3; Table 4). This reduction in MCAv compared to daytime values has been previously discussed (Hajak et al. 1994; Klingelhöfer 2012; Kuboyama et al. 1997) and predominantly seems to take place during slow-wave (deep) sleep. Interestingly, EtCO_2_, which is typically a powerful modulator of cerebral blood flow, remained unchanged during sleep. However, previous studies have shown that CO_2_ sensitivity is reduced with sleep (Meadows et al. 2003), which could explain why MCAv decreases despite stable EtCO_2_. Instead, the driver for decreased brain blood flow during sleep is likely to be brain metabolism. Positron emission tomography studies (Buchsbaum et al. 1989; Maquet et al. 1990) have demonstrated metabolism to be decreased during slow-wave sleep, thereby lowering oxygen and nutrient consumption, which may cause blood flow to the brain to decrease. However, while slow-wave sleep shows decreased metabolism, rapid eye movement (REM) sleep has levels almost identical to awake states. As the duration and incidence of REM sleep typically increases over time spent asleep (most REM phases occurring at the end of the night), this aligns with increases in MCAv that we found as the night progressed: from the lowest values around the 4th hour asleep (43 ± 8 cm·s^− 1^), MCAv increased to 47 ± 9 cm·s^− 1^ shortly before waking up (hours 6- awake). While speculative, this may indicate that the latter values were taken during REM sleep.

In the current study, PCA velocity and conductance (as a marker of changes in vascular tone) were consistent throughout the day. While we did not measure these parameters at night, choroidal thickness has been investigated for diurnal variations in the past, and studies in chickens and marmosets with night-time measurements revealed peak choroidal thickness around midnight and a minimum around noon (Nickla 2006; Nickla et al. 2002; Papastergiou et al. 1998). These variations have been confirmed by studies in humans, where peak thickness occurred in the late evenings (Chakraborty et al. 2011) or early nighttime (Ostrin et al. 2019). Interestingly, intraocular pressure fluctuates in a similar pattern, although with opposite peaks (Ostrin et al. 2019). Similarly, both ICP and central venous pressure (CVP) decrease over night (Lawley et al. 2017) compared to their supine values.

A nighttime peak thickness of the choroid may be in accordance with blood flow to the eye being higher during sleep than in daytime. Any nighttime changes are likely to happen due to sleep and/or a circadian rhythm, as PCA conductance remaining stable throughout posture changes suggests there not to be a postural effect. However, choroidal thickness is impacted by bed rest, increasing significantly after 24 h of resting in a head-down tilt position (Lawley et al. 2020). While short-termed changes in posture have no effect on blood flow to the eye, it is possible that bed rest and therefore potentially spaceflight induces further reaching changes.

### Effects of lower-body negative pressure on eye blood flow

Choroid engorgement is a prominent finding of SANS and is associated to spaceflight as well as bed rest studies. Recently, low-level LBNP has been shown to attenuate choroid engorgement associated with short-term head down tilt and supine bed rest (Hearon et al. 2022; Lawley et al. 2020) – an effect observed hours after the application of LBNP was stopped. By creating a negative vacuum on the lower body, the LBNP technique attenuates cephalad fluid shifts associated with the supine posture (Wolthuis et al. 1974), including a reduction in central venous (Mack et al. 1987, 1993) and intracranial (Lawley et al. 2020; Petersen et al. 2019) pressure.

We initially proposed the protective mechanism through which LBNP attenuates choroid engorgement during bed rest to be a reduction in blood flow to the eye. However, PCAv was slightly elevated and PCAv conductance was unaltered in the LBNP protocol (see Table 5). The increase of PCAv was primarily due to the slight increase in blood pressure (i.e. perfusion pressure) associated with LBNP, thus while future studies could look at longer durations of LBNP, its protective role in reducing arterial inflow and vascular volume is unlikely. However, Petersen and colleagues have shown that LBNP reduces cerebrospinal fluid pressure on the brain (Petersen et al. 2019), and Arbeille and colleagues have noticed a spaceflight-induced increase in middle cerebral *vein* velocity, suggesting that the fluid shift impacts cerebral venous circulation. The vein velocity increase was restored to preflight values by the application of low-level LBNP (Arbeille et al. 2021). Future research could examine if changes in choroidal venous volume and its changes with posture and LBNP are due to manipulation of the intrachoroid venous volume.

### Limitations

We did not have the means of measuring ICP and IOP in the current study, and therefore used values taken from literature. We took care to include ICP values which were measured invasively and from healthy participants only. However, no values for the prone or lateral positions were found. While we assume ICP in the lateral position to be similar to measurements taken in a supine one, this may not apply for the prone posture. The prone position introduces multiple cardiopulmonary changes which may influence ICP, including a slight hypoventilation-induced hypercapnia, and increased central venous pressure (CVP) from elevated intrathoracic and intra-abdominal pressure. Hypercapnia and elevated CVP may result in a Valsalva-like state, elevating ICP (Lueck and McClelland 2019). This elevation seems to be exacerbated by obesity and as all our participants were lean (mean body mass index 23 ± 2 kg·cm^− 2^), we assume ICP elevations in the prone position, if present, to be minimal compared to supine values.

The fact that we applied LBNP only for short duration may have caused the pressure-passive increase in PCA velocity. However, they confirm the choroid’s imperfect ability to autoregulate as despite a slight increase in blood pressure, MCAv remained stable due to a decrease in cerebral vascular conductance. Long duration studies, preferably in space, will be necessary to determine if LBNP can be used as an effective countermeasure against SANS and its underlying mechanisms.

### Conclusion

The primary finding of the current study is that there are several major hemodynamic changes to the brain and eye that occur when changing from the upright to supine posture (Gx axis). In contrast, rotating around the Gz axis had little impact on most physiological systems. Likewise, waking hours (~ 8 a.m. – 5 p.m.) had minor effects of the cardiovascular system, whereas sleep caused a prominent reduction in both blood pressure and blood flow to the brain. Importantly, correcting for tissue weight and each of the hydrostatic gradients is critical to accurately describe changes in downstream vascular tone associated with changes in posture. Finally, low-level LBNP had little impact on arterial blood flow to the eye, limiting it as a mechanism for the protective effect on choroidal thickness.

## Abbreviations

ANOVA: Analysis of variance
CBF: Cerebral blood flow
CVP: Central venous pressure
EAM: External auditory meatus
ECG: Electrocardiogram
EtCO2: End-tidal carbon dioxide
ICA: Internal carotid artery
IOP: Intraocular pressure
ICP: Intracranial pressure
LBNP: Lower-body negative pressure
MAP: Mean arterial pressure
MCA: Middle cerebral artery
PCA: Posterior ciliary artery
PLC: Posterior lamina cribrosa
REM: Rapid eye movement
SANS: Spaceflight-Associated Neuro-Ocular Syndrome
VA: Vertebral artery
